# The contribution of moderation foods to total energy intake by sociodemographic characteristics in U.S. adults: National Health and Nutrition Examination Survey, 2017 – 2020

**DOI:** 10.1101/2025.09.30.25336983

**Published:** 2025-10-02

**Authors:** Leah M. Lipsky, Amara Channel-Doig, Tonja R. Nansel

**Affiliations:** Social and Behavioral Sciences Branch, Division of Population Health Research, *Eunice Kennedy Shriver* National Institute of Child Health and Human Development, Bethesda, MD.

## Abstract

**Background:**

Intake of foods high in nutrients of concern (moderation foods) contribute a large proportion of total energy intake in the U.S, but little is known about how moderation food intake varies across sociodemographic subgroups.

**Objective:**

This observational study assessed sociodemographic differences in the contribution of moderation food to energy intake in U.S. adults.

**Methods:**

Data from 24-hour dietary recalls of participants 20 – 80 years of age in the 2017–2020 National Health and Nutrition Examination Survey (n=7706) were used to calculate the percent of energy intake from foods meeting any of the following criteria: added sugar >20% energy, sodium >460mg per serving, refined grains >50% of total grains or >10:1 ratio of carbohydrate to fiber content, saturated fat >20% energy, total fat >9% by weight (applied only to vegetables, sweets, and snacks), and all alcoholic beverages. Moderation food intake was compared by age, sex, education, income poverty ratio (IPR), and race/ethnicity, and linear regressions with multiplicative interaction terms investigated whether race/ethnicity or education modified relationships of IPR with moderation food intake.

**Results:**

Mean moderation food contributed 75% (95%CI:75%–77%) of energy intake in U.S. adults, and was higher in younger (mean=79%) vs. older (mean=72%–75%) adults, males (mean=77%) vs. females (mean=74%), and adults in the lowest (mean=79%) vs. highest (mean=74%) income quartile. Moderation food intake was lowest in Non-Hispanic Asian adults (mean=66%) versus other race/ethnicity groups (m=73% - 80%) and in the highest educated (mean=71%) vs. less-educated adults (m=75%–79%). The relationship of income with moderation food intake did not differ by race/ethnicity overall, but the relationship was significantly more negative for non-Hispanic Black adults than in Mexican American/Hispanic adults. Similarly, while the relationship of income with moderation food intake did not differ overall by education, marginal slopes estimates indicated an inverse association among high school graduates (second highest education group; β = −1.1 95%CI = −2.1 to −0.2) and null associations for all other adults.

**Conclusions:**

High moderation food intake across all U.S. adult subgroups suggests a need for population-wide changes in food selection to meet dietary guidelines.

## Introduction

The 2020–2025 Dietary Guidelines for Americans (DGA) recommend limiting intake of added sugar, saturated fat, sodium, refined grains, and alcoholic beverages (“moderation components”) due to their associations with multiple adverse health outcomes and their tendency to displace intake of foods that provide essential nutrients (1, 2). However, most Americans exceed recommended maximum intakes of refined grains (>90%), added sugar (70%), saturated fat (70%), and sodium (90%) contributing to widespread increased risk for chronic diseases (1).

Sociodemographic differences in overall diet quality have been reported in multiple studies. Analyses of recent NHANES data show modestly higher overall diet quality (as indicated by Healthy Eating Index-2015 scores) in females (mean HEI=60) versus males (mean HEI=56), non-Hispanic Asian (mean HEI=65) and Hispanic (mean HEI=58) adults versus non-Hispanic white (mean HEI=57) and non-Hispanic black (mean HEI=54) adults, and in adults with household income greater than 350% of the federal poverty level (mean HEI=60) versus those with incomes less than 350% of the federal poverty level (mean HEI=56) (3). Education is also positively associated with better overall diet quality (4–8), and some evidence suggests that associations of income with diet quality differ by education and race/ethnicity (8, 9). In the U.S., educational attainment is positively correlated with income (10), and both income and education differ by race/ethnicity (11, 12). However, few previous studies on sociodemographic correlates of diet quality have examined the potential for confounding or interactions between these variables.

Research on sociodemographic differences in intake of moderation dietary components has focused on individual nutrients or food groups (such as sugar-sweetened beverages or snack foods) (13–16), ultra-processed foods (based on processing rather than nutritional components) (4, 17, 18), or “junk” (19) or “fast” (20) foods defined in multiple ways and applied only to select food groups. Given the lack of a guidelines-based definition for foods high in nutrients of concern that can be applied to all foods in the Food and Nutrient Database for Dietary Studies (FNDDS), we previously developed a method to identify moderation foods based on thresholds for added sugar, sodium, fat, refined grains, and alcohol, which effectively distinguished between foods with high versus low nutrient density (21). Examining the distribution of moderation food intake across population subgroups using a systematic, guidelines-based indictor will help clarify how moderation food contributes to socioeconomic differences in overall diet quality and identify subgroups who may benefit most from targeted interventions. This study aimed to compare moderation food intake across sociodemographic characteristics among U.S. adults and investigate whether education or race/ethnicity modifies associations of income with moderation food intake.

## Methods

### Study design and population

This study used publicly available data from the pre-pandemic cycle (2017 – 2020) of the National Health and Nutrition Examination Survey (NHANES), administered by the National Center for Health Statistics (NCHS). NHANES is a population-based, cross-sectional survey that uses a complex, stratified, multistage probability sample design to generate a representative sample of the civilian, non-institutionalized U.S. population. Details on the study design, protocol, and data collection have been documented elsewhere (22). The protocol was approved by the NCHS Research Ethics Review Board, and informed consent was obtained from participants ≥ 18 years of age.

The analytic sample for this study included adults at least 20 years old with at least one 24-hour dietary recall determined to be reliable by NCHS (23), and data on at least one of the sociodemographic variables. Of the n = 9,232 total adult participants, n = 688 had no dietary recall data and n = 837 had no valid recalls. The final analytic data set included n = 7707 participants. This secondary analysis of deidentified publicly available data was exempt from institutional review board review.

### Data collection

Participants self-reported sociodemographic characteristics during an in-home interview administered by trained personnel. Dietary intake data come from the What We Eat In America dietary interview component of NHANES; trained interviewers conducted two 24-hour dietary recalls using the USDA’s Automated Multiple-Pass Method (24), with the first recall administered in-person at the mobile exam center and the second by telephone approximately 3 – 10 days after the initial recall.

### Moderation food intake

Foods reported in the 24-hour diet recalls were classified as moderation foods if they met any of the previously established criteria: added sugar >20% of energy; sodium >460mg per serving; refined grains >50% of total grains and >10:1 carbohydrate: fiber ratio by weight; saturated fat >20% of energy (excluding vegetables); total fat >9% by weight (applied only to vegetables, sweets, and snacks), and all alcoholic beverages (25). Foods that did not meet any criteria were classified as non-moderation. Total moderation food intake (% kcal) was calculated from the sum of energy intake from moderation foods relative to total energy intake across both diet recalls.

### Sociodemographic characteristics

NHANES categorizes age as 20 – 39 y, 40 – 59 y, and ≥ 60 y (26). Race/ethnicity is categorized as Mexican American/Hispanic, non-Hispanic white, non-Hispanic black, non-Hispanic Asian, and multi/other. Education is categorized as less than high school, high school degree/general equivalency diploma, some college, college graduate or above. Income poverty ratio (IPR) (27) is a continuous measure that reflects household income relative to the federal poverty threshold, accounting for household size and composition. NHANES truncates the maximum IPR at 5 due to disclosure concerns; IPR quartiles were used for analysis.

### Statistical analysis

All analyses used survey methods to account for the complex sampling design of NHANES using StataSE version 18. Frequencies and percentages were calculated for sociodemographic characteristics, and means and 95% confidence intervals of moderation food intake (% kcal) were calculated for each sociodemographic subgroup. Differences in mean moderation food intake by sociodemographic characteristics were evaluated using linear regressions and post-estimation pairwise comparisons; Sidak adjustment was used to account for multiple comparisons. We used separate models with multiplicative interaction terms to investigate whether education or race/ethnicity modified associations of IPR (examined as a continuous variable) with moderation food intake.

## Results

The sample was approximately evenly split according to age group and sex (Table 1). Sixty-three percent of the sample was non-Hispanic white. Ten percent of the weighted sample earned less than a high school degree, and the rest of the sample was approximately evenly split between education groups. IPR was disproportionately concentrated at the highest truncated value (5), with the mean ± SD of the upper quartile = 4.9±0.1.

**Figure 1.**
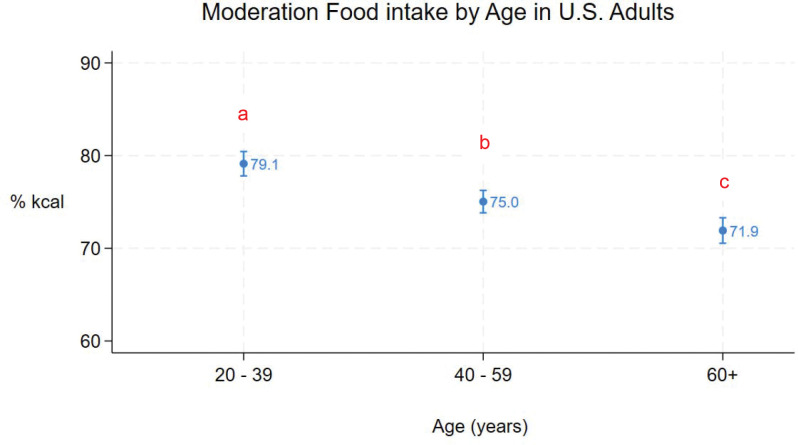
Moderation food intake (% kcal) in U.S. adults by age (means and 95%CIs). Different subscripts denote statistically different pairwise comparisons (using Sidak adjustment for multiple comparisons).

**Figure 2.**
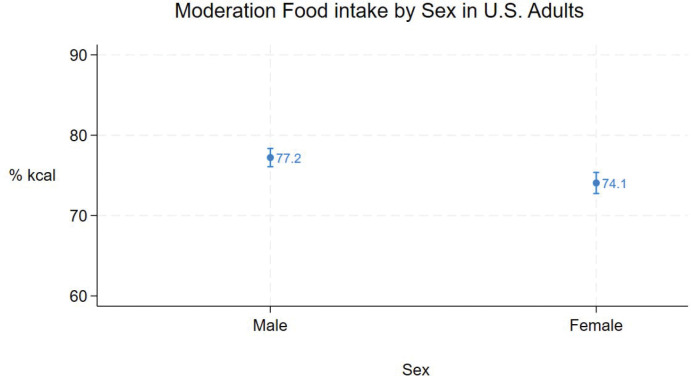
Moderation food intake (% kcal) in U.S. adults by sex (means and 95%CIs).

**Figure 3.**
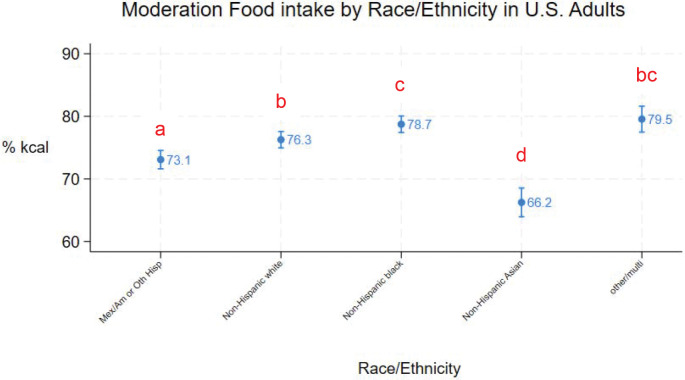
Moderation food intake (% kcal) in U.S. adults by race/ethnicity (means and 95%CIs). Different subscripts denote statistically different pairwise comparisons (using Sidak adjustment for multiple comparisons).

**Figure 4.**
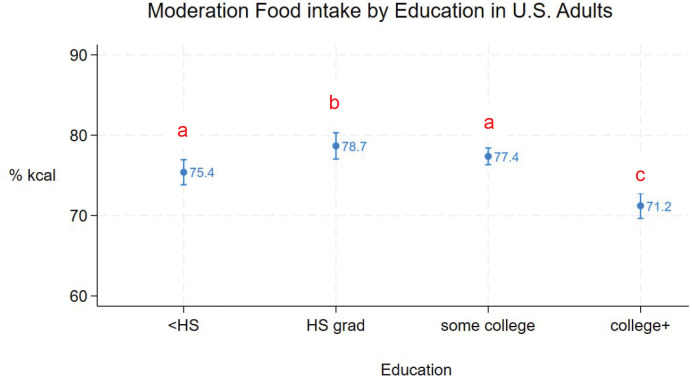
Moderation food intake (% kcal) in U.S. adults by education (means and 95%CIs). Different subscripts denote statistically different pairwise comparisons (using Sidak adjustment for multiple comparisons).

**Figure 5.**
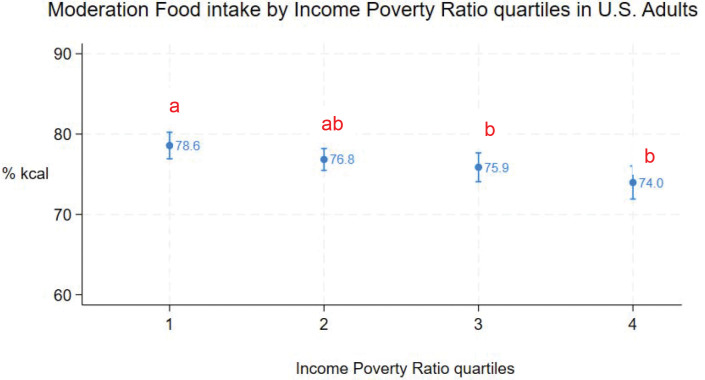
Moderation food intake (% kcal) in U.S. adults by income poverty ratio quartiles (means and 95%CIs). Different subscripts denote statistically different pairwise comparisons (using Sidak adjustment for multiple comparisons).

Mean moderation food intake for all adults was 75% (95% CI = 75% - 77%). Figures 1 – 5 show mean (95% CI) moderation food intake (% kcal) by sociodemographic characteristics. Pairwise comparisons of mean moderation food intake by sociodemographic characteristics are shown in Table 2. Group mean differences were generally < 5 percentage points. The largest mean differences (>10 points) were observed between race/ethnic groups, particularly when comparing non-Hispanic Asian adults versus non-Hispanic white, non-Hispanic black, and other/multi adults. Pairwise comparisons between other race/ethnic groups, income-poverty ratio quartiles, and between sexes were <5 points. Comparisons between age and education groups were < 10 points.

Mean moderation food intake decreased with age, ranging from 79% (95%CI: 78% - 80%) in adults 20 – 39 years of age to 72% (95%CI: 71% - 73%) in adults ≥ 60 years of age (p < 0.001 for all pairwise comparisons), and was higher in males than females (Figure 2, Table 2).

Mean moderation food intake was lower in non-Hispanic Asian adults (mean=66%, 95%CI: 64% - 69%) than adults in other racial/ethnic groups and highest in multi/other (mean=80%, 95%CI: 77% - 82%) and non-Hispanic black adults (mean=79%, 95%CI: 77% - 80%) (Figure 3, Table 2). Moderation food intake was lower in Mexican American/Hispanic adults (mean=73%, 95%CI: 72% - 75%) than in non-Hispanic white adults (mean=76%, 95%CI: 75% - 78%). Mean moderation food intake in adults with multi/other race/ethnicity was not statistically different than in non-Hispanic white (p=0.08) or black (p=0.998) adults. All other pairwise differences by race/ethnicity were statistically significant (p≤0.02).

Mean moderation food intake in adults with at least a college degree was lower than in those with less education (Figure 4, Table 2). Mean moderation food intake in adults with some college education was not different from in those with a high school degree or less (Figure 4, Table 2).

Mean moderation food intake was inversely associated with IPR quartile (Figure 5, Table 2). However, only the pairwise differences between the 1^st^ and 3^rd^ (76%, 95%CI: 74% - 78%) quartiles and 1^st^ and 4^th^ quartiles were statistically significant.

**Figure 6.**
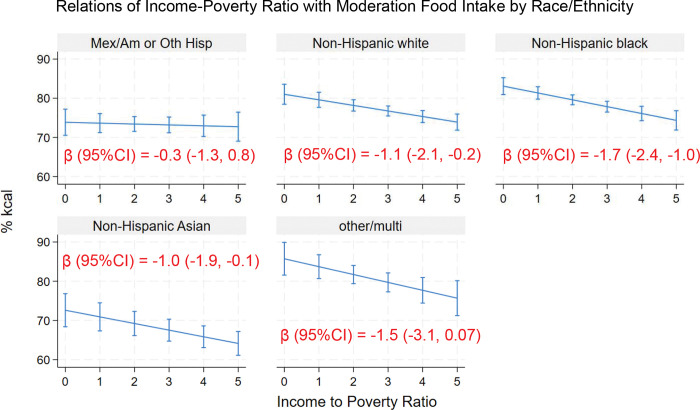
Relationship of income with moderation food intake (% kcal) in U.S. adults by education (means and 95%CIs). Coefficient estimates for the marginal associations of income poverty ratio with moderation food intake (%kcal) by education are shown.

Income was inversely associated with moderation food intake in all race/ethnic groups except Mexican American/Hispanic and other/multi adults (Figure 6). Results from the adjusted Wald test (F(4, 22) = 1.56, Prob > F = 0.22) indicated no statistically significant interaction overall between IPR and race/ethnicity in relation to moderation food intake. However, the coefficient estimate for non-Hispanic Black adults was significantly more negative than that for Mexican American/Hispanic adults (Table 3).

**Figure 7.**
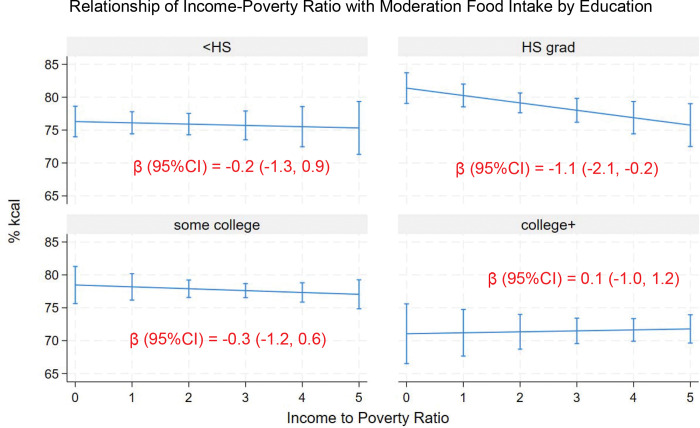
Relationship of income to poverty ratio with moderation food intake (% kcal) in U.S. adults by race/ethnicity (means and 95%CIs). Coefficient estimates for the marginal associations of income poverty ratio with moderation food intake (% kcal) by race/ethnicity are shown.

Income was inversely associated with moderation food intake in high school graduates only (Figure 7). There was no statistically significant overall interaction between IPR and education in relation to moderation food intake (F(3, 23) = 2.6, Prob > F = 0.08) (Table 3). The association was modestly more negative in high school graduates, but the estimate was not statistically different from the referent group.

## Discussion

Mean moderation food intake ranged from 70% - 80% of energy intake across most segments of the U.S. adult population in 2017 – 2020. While some statistically significant differences were attributable to sex, age, income, race/ethnicity, and education, all subgroups obtained the majority of energy intake from moderation food, and the only subgroup with mean moderation food intake less than 70% kcal was non-Hispanic Asian adults (66%). Moderation food intake differed more by race/ethnicity and education than other socioemographic characteristics, and associations of income with moderation food intake did not vary by race or education.

Our findings are consistent with previous studies that reported small but statistically significant socioeconomic disparities in adult diet quality (4, 18, 28–33). In those studies, diet quality scores across all subgroups were far from optimal, with mean group differences ranging from <1% to 9%. Reports have often emphasized statistically significant differences while giving less attention to their small magnitudes, potentially contributing to the misconception that low diet quality affects only specific population subgroups. In contrast, these findings indicate that suboptimal dietary patterns are widespread, regardless of sex, income, race/ethnicity, or education, despite some statistically significant differences. While the DGA recommend that 85% of daily energy intake come from nutrient-dense foods, leaving only 15% for added sugars, fats, or alcohol (1), these findings suggest that dietary behaviors across all sociodemographic subgroups of U.S. adults are essentially reversed, with moderation food accounting for at least two-thirds of energy intake and non-moderation foods contributing only 20% to 30%. The magnitudes of subgroup differences were smallest by income-poverty ratio (1 – 4 points) and sex (3 points), and largest by age (8 points between the youngest and oldest adults) and race/ethnicity (7 – 13 points between non-Hispanic Asians versus other groups).

The consistently high moderation food intake observed across different sociodemographic subgroups suggests that sociodemographic factors may not be primary drivers of low diet quality in U.S. adults. This aligns with findings from the Consumer Expenditure Survey, which demonstrate that, across all income groups, the largest share of food-at-home spending (35%) goes to miscellaneous foods (e.g. frozen meals, snacks, canned and packaged soups, potato chips, condiments, seasonings), while only 20% - 25% goes to meat, poultry, fish, and eggs; and only 19% to fruits and vegetables (34). These purchasing patterns suggest that households may prioritize spending on foods for convenience, social, cultural, emotional, or other purposes (35–37), rather than adherence to dietary guidelines. Additionally, while food accessibility is a barrier to healthy eating, national data indicate that 6.1% of the U.S. population lives in areas with limited supermarket access, with only 1.7% of households being both far from a foodstore and without vehicle access (38).

The DGA focus on nutrients rather than foods to limit may have inadvertently weakened its effectiveness in discouraging intake of moderation foods in the U.S population. Since the 1970s, guidelines related to decreasing intakes have provided nutrient-based rather than food-based recommendations, a practice influenced by industry pressures (39) and enabled by the closed nature of DGA meetings prior to the 2010 – 2015 cycle. Nutrient-based guidelines are more difficult for consumers to understand and implement than food-based recommendations (40), partly since nutrient-based guidelines require viewing and correctly interpreting nutrition labels that present nutrients per serving, which consumers must then accurately extrapolate to their total daily intake. Consequently, consumers may be unaware when intake of nutrients of concern exceeds recommended amounts. Although the shift to public DGA Committee meetings beginning with the 2010 – 2015 cycle has offered the opportunity to incorporate food-based recommendations for moderation components, the nutrient-based approach established during previous closed meetings has persisted.

By contrast, the Chilean government adopted thresholds to classify packaged foods high in kcal, sugar, sodium, or saturated fat, and applied clear front-of-package labels along with restrictions on school food and marketing to children. These policies significantly reduced consumer purchases and industry supply of high-sugar and high-sodium products, while also decreasing unhealthy marketing to children, limiting availability in schools, and improving consumers’ ability to identify and avoid unhealthy foods (41–46). Collectively, this evidence demonstrates how, even though individual food choices are motivated by complex social, cultural, and emotional factors, policies that influence food environments can affect dietary behaviors. Furthermore, it illustrates how regulatory approaches that use systematic food classification based on nutrient thresholds can drive meaningful changes in the food environment and serve as a powerful tool to improve diet quality and reduce chronic disease risk at the population level.

Several elements of this study strengthen the internal and external validity of the findings. The study used data from a large, nationally representative sample of the U.S. population that included administration of two dietary recalls using the Automated Multi-Pass Method. One limitation is that the use of self-reported dietary intake is susceptible to reporting bias that may weaken internal validity. However, we combined four years of NHANES data and used analytic methods to obtain reliable and generalizable estimates of overall intake of moderation food for multiple sociodemographic subgroups. We also examined potential confounding and effect modification between correlated characteristics (income, education, and race/ethnicity), which provides more valid estimates than bivariate analyses. As this was an observational study, it was descriptive rather than hypothesizing causal relationships.

## Conclusion

Moderation food intake is high in U.S. adults across multiple sociodemographic characteristics, indicating an urgent, population-wide need to replace moderation food with core foods that are aligned with nutritional requirements. The moderation food classification method may facilitate moving towards more explicit, food-based guidance and policy changes that modify the food environment to improve diet quality and chronic disease outcomes at the population level.

## Figures and Tables

**Table 1.** Sample characteristics (n = 7707)

Characteristic	Adults (≥ 20 y)	
	N	%

Age, years		
20 – 39	2358	36
40 – 59	2548	34
60+	2801	30
Gender		
Male	3745	48
Female	3962	52
Race/ethnicity		
Mexican American/Hispanic	1669	16
Non-Hispanic White	2758	63
Non-Hispanic Black	2071	11
Non-Hispanic Asian	841	6
Multi/other	368	4
Education		
Some high school	1358	10
High school graduate	1863	28
Some college	2567	31
College graduate or more	1910	32
Income poverty ratio quartile		
1 (mean ± SD = 0.7 ± 0.4)	1689	17
2 (mean ± SD = 1.8 ± 0.3)	1685	19
3 (mean ± SD = 3.3 ± 0.5)	1690	27
4 (mean ± SD = 4.9 ± 0.1)	1672	37

**Table 2.** Sidak-adjusted pairwise comparisons of mean moderation food intake (% kcal) in U.S. adults by sociodemograpic characteristics (National Health and Nutrition Examination Survey, 2017 – 2020)

Characteristic	Comparison	Mean difference (% kcal)	95% CI	*p* - value

Age, y	40 – 59 vs 20 – 39	−4.1	−5.9, −2.3	<0.001
	>=60 vs 20 – 39	−7.2	−8.8, −5.6	<0.001
	>=60 vs 40 – 59	−3.1	−4.8, −1.5	<0.001
Sex	Female vs Male	−3.2	−4.4, −1.9	<0.001
Race/ethnicity	NH white vs Mex/Am Hisp	3.2	0.4, 6.0	0.02
	NH black vs Mex/Am Hisp	5.6	2.9, 8.4	<0.001
	NH Asian vs Mex/Am Hisp	−6.9	−10.5, −3.2	<0.001
	other/multi vs Mex/Am Hisp	6.4	2.6, 10.3	<0.001
	NH black vs NH white	2.5	0.4, 4.6	0.01
	NH Asian vs NH white	−10.0	−13.2, −6.8	<0.001
	other/multi vs NH white	3.3	−2.5, 6.8	0.08
	NH Asian vs NH black	−12.5	−16.9, −8.1	<0.001
	other/multi vs NH black	0.8	−2.6, 4.2	0.998
	other/multi vs NH Asian	13.3	8.7, 17.9	<0.001
Education	HS grad vs. < HS	3.3	0.7, 5.9	0.008
	Some college vs < HS	2.0	−0.6, 4.6	0.22
	College graduate vs < HS	−4.2	−6.9, −1.5	0.001
	Some college vs HS grad	−1.3	−3.4, 0.8	0.45
	College graduate vs HS grad	−7.5	−10.7, −4.2	<0.001
	College graduate vs some college	−6.2	−8.5, −3.8	<0.001
Income-poverty ratio quartiles	2 vs 1	−1.8	−4.7, 1.2	0.47
	3 vs 1	−2.7	−5.9, −0.4	0.02
	4 vs 1	−4.6	−8.1, −1.2	0.005
	3 vs 2	−1.0	−3.7, 1.8	0.91
	4 vs 2	−2.9	−6.6, 0.9	0.21
	4 vs 3	−1.9	−5.6, 1.8	0.63

Abbreviations: NH − non-Hispanic, Mex/Am Hisp − Mexican American or other Hispanic, HS − high school

**Table 3.** Coefficient estimates from regression models estimating interactions of income poverty ratio (IPR) with race/ethnicity and education on moderation food intake (% kcal) in U.S. adults.

Model	Independent variable	β	95% CI	*P*-value

Race/ethnicity	IPR	−0.3	−1.3, 0.7	0.57
	Race/ethnicity			
	Mexican American/Hispanic		REF	
	Non-Hispanic White	6.1	2.1, 10.1	0.005
	Non-Hispanic Black	8.6	5.4, 11.8	<0.001
	Non-Hispanic Asian	−3.6	−7.6, 0.4	0.08
	Other/multi	9.3	3.5, 15.1	0.003
	Interaction of IPR with race/ethnicity			
	Mexican American/Hispanic		REF	
	Non-Hispanic White	−0.9	−2.2, 0.5	0.21
	Non-Hispanic Black	−1.4	−2.6, −0.3	0.02
	Non-Hispanic Asian	−0.7	−1.9, 0.4	0.21
	Other/multi	−1.2	−3.5, 1.1	0.28
	Intercept	74.1	71.4, 76.8	<0.001
Education	IPR	0.1	−1.0, 1.2	0.79
	Education			
	Less than high school	5.2	0.8, 9.7	0.02
	High school graduate	10.3	5.7, 14.9	<0.001
	Some college	7.4	3.0, 11.8	0.002
	College graduate or more		REF	
	Interaction of IPR with education			
	Less than high school degree	−0.3	−1.8, 1.1	0.63
	High school graduate	−1.3	−2.6, 0.02	0.05
	Some college	−0.4	−1.5, 0.7	0.43
	College graduate or more		REF	
	Intercept	71.1	66.5, 75.6	<0.001

## Abbreviations:

HEI: Healthy Eating Index
IPR: Income-Poverty Eatio
NHANES: National Health and Nutrition Examination Survey
NCHS: National Center for Health Statistics
